# Comparative Clinical Effectiveness of Intra-Articular Leukocyte-Rich Platelet-Rich Plasma and Hyaluronic Acid in Treating Knee Osteoarthritis Pain: An Updated Systematic Review and Meta-Analysis of Randomized Controlled Trials

**DOI:** 10.1016/j.arrct.2025.100573

**Published:** 2026-01-28

**Authors:** Yu-Ning Peng, Di Huang, I-Ling Chou, Henry L. Lew

**Affiliations:** a Department of Physical Medicine & Rehabilitation, Chang Gung Memorial Hospital, Taoyuan, Taiwan; b Division of Clinical and Translational Research, McGill University, Montreal, Québec, Canada; c Hualien Tzu Chi Hospital, Buddhist Tzu Chi Medical Foundation, Hualien City, Taiwan; d Department of Epidemiology, Johns Hopkins Bloomberg School of Public Health, Baltimore, MD; e Department of Medical Education, China Medical University, Taichung, Taiwan; f Department of Communication Sciences and Disorders, John A. Burns School of Medicine, University of Hawaii, Honolulu, HI; g Department of Physical Medicine and Rehabilitation, Virginia Commonwealth University, Richmond, VA; h Department of Medical Education, Office of Medical Education, John A. Burns School of Medicine, University of Hawaii, Honolulu, HI

**Keywords:** Hyaluronic acid, Knee, Osteoarthritis, Pain management, Platelet-rich plasma, Rehabilitation

## Abstract

**Objective:**

To investigate the clinical effectiveness of intra-articular leukocyte-rich-platelet-rich plasma (LR-PRP) injections versus hyaluronic acid (HA) for pain management in patients with knee osteoarthritis (OA).

**Data Sources:**

Four electronic databsases were searched, including PubMed, EMBASE, Scopus, and Web of Science. Studies included in our analysis were published between November 1, 2011, and July 14, 2023.

**Study Selection:**

Randomized controlled trials (RCTs) that compared intra-articular LR-PRP versus HA injections in human adults diagnosed with knee OA.

**Data Extraction:**

The Cochrane Risk of Bias Tool was used to assess the risk of bias and methodological quality of included RCTs. The mean differences (MDs) and SDs with 95% CIs were used to compare continuous outcomes, using the Review Manager 5.4 software. Two reviewers independently extracted data on patient demographics and study outcome, including Western Ontario and McMaster Universities Osteoarthritis Index (WOMAC) scores, Visual Analog Scale scores, and adverse events.

**Data Synthesis:**

Nineteen RCTs involving 1771 patients were included. LR-PRP demonstrated significant pain relief compared with HA at 3 months posttreatment, but not at 1, 6, or 12 months. At 12 months, LR-PRP was associated with improved WOMAC physical function (MD=−6.37; 95% CI, −12.51 to −0.24; *I*²=85%) and stiffness scores (MD=−0.63; 95% CI, −0.98 to −0.27; *I*²=0%), but no differences were observed at earlier timepoints or in total WOMAC scores. Adverse event rates were similar between groups.

**Conclusions:**

LR-PRP provides long-term functional improvements in patients with chronic knee OA, though evidence for short-term functional benefits remains limited. LR-PRP shows no long-term advantage over intra-articular HA injections in pain relief. Overall, the evidence does not support the superiority of LR-PRP over HA in managing knee OA.

Knee osteoarthritis (OA) is a prevalent condition that greatly affects physical function and overall quality of life.^1^ Nonsurgical treatments, including intra-articular injections and exercise rehabilitation programs, are essential for symptom management and delaying the need for total knee arthroplasty.^2^ Intra-articular injections of platelet-rich plasma (PRP), hyaluronic acid (HA), and corticosteroids are commonly used for knee OA.^3^^,^^4^ Among these, intra-articular HA injections have demonstrated moderate efficacy in relieving pain and improving function in patients with mild to moderate knee OA, particularly during the early postinjection period. These effects are attributed to enhanced joint lubrication and viscoelasticity, potentially delaying the need for surgical intervention.^5^, ^6^, ^7^ HA enhances the viscoelastic properties of synovial fluid, thereby improving joint lubrication and mechanical function in patients with knee OA.^8^ However, its clinical utility remains a subject of debate. Notably, the 2021 Clinical Practice Guidelines from the American Academy of Orthopedic Surgeons^9^ recommend against the use of HA for symptomatic knee OA because of inconsistent evidence regarding its efficacy. Despite this, HA remains a common comparator in clinical trials evaluating novel therapies.

PRP, a blood-derived product enriched with growth factors, has demonstrated greater potential for providing sustained pain relief and improving functional outcomes.^10^ PRP is categorized into leukocyte-poor PRP (LP-PRP) and leukocyte-rich PRP (LR-PRP), distinguished by their leukocyte and platelet profiles. LP-PRP typically contains a comparable or slightly lower platelet concentration than LR-PRP but is defined by minimal leukocyte content, making it suitable for conditions in which reducing inflammation is paramount, such as acute injuries or postsurgical recovery.^11^ In contrast, LR-PRP is characterized by higher leukocyte concentrations, including neutrophils and monocytes, as well as elevated platelet levels, which may enhance tissue repair through immune-mediated pathways but can also elicit stronger inflammatory responses.^12^ Although the precise mechanism remains under investigation, the therapeutic potential of LR-PRP is hypothesized to stem from the release of supraphysiological concentrations of growth factors, such as transforming growth factor-beta and platelet-derived growth factor.^13^ Furthermore, although leukocytes may induce transient inflammation, macrophages play a crucial role in tissue remodeling and immune modulation, potentially facilitating the transition from the inflammatory phase to the reparative phase in chronic OA pathology.^14^ Clinically, LP-PRP is preferred for promoting healing with minimal inflammation, such as in OA or soft tissue recovery^15^, whereas LR-PRP is often more effective for chronic conditions, like tendinopathy, in which a controlled inflammatory response may stimulate repair.^16^ The choice between LP-PRP and LR-PRP depends on the therapeutic context, underscoring the need for standardized protocols to guide their application.

Several randomized controlled trials (RCTs) have demonstrated that LR-PRP provides greater benefits than HA in treating knee OA, concluding that LR-PRP significantly outperformed HA in achieving clinically significant pain relief^17^ and led to better Western Ontario and McMaster Universities Osteoarthritis Index (WOMAC) total and pain scores compared with HA at 6 months posttreatment.^18^ Nevertheless, multiple RCTs have reported no notable advantage of LR-PRP compared with HA. A double-blinded RCT by Filardo et al^19^ found that LR-PRP and HA yielded comparable outcomes in pain relief and functional improvement postinjection. Di Martino et al^20^ confirmed these results, showing no significant differences between LR-PRP and HA in clinical outcomes. These consistent findings across RCTs underscore the need for further research to identify optimal treatment protocols for knee OA management. A meta-analysis found that PRP, including LR-PRP, generally outperformed HA in improving clinical outcomes like pain and function, as assessed by WOMAC and Visual Analog Scale (VAS) scores; however, it noted that LP-PRP might be more effective than LR-PRP, highlighting variability in PRP formulations.^21^ Another meta-analysis suggested that PRP, regardless of leukocyte content, consistently provided better pain relief and functional improvement than HA.^22^ Therefore, more extensive and methodologically rigorous meta-analyses are crucial to clarify the comparative effectiveness of LR-PRP versus HA and resolve the inconsistencies observed in the management of knee OA.

To address these gaps in evidence, this systematic review and meta-analysis focuses exclusively on RCTs that adhere to stringent methodological standards and consistent inclusion criteria. By synthesizing high-quality evidence, our goal is to provide a more precise understanding of the efficacy of intra-articular LR-PRP injections in comparison to HA for pain management in patients with knee OA.

## Methods

This systematic review was conducted following the guidelines outlined in the Preferred Reporting Items for Systematic Reviews and Meta-Analyses statement^23^ and the guidelines outlined in the Cochrane Handbook for Systematic Reviews of Interventions.^24^ This study was registered in PROSPERO (CRD420251091050). Institutional Review Board approval was not required, as the study exclusively analyzed data from previously published RCTs without any additional handling of individual patient information.

### Inclusion and exclusion criteria

We limited our search to RCTs conducted in humans. The inclusion criteria for this study were as follows: (1) RCTs comparing intra-articular LR-PRP injections with HA injections; (2) adult participants (aged ≥18y) diagnosed with knee OA pain; and (3) studies published in English. Studies were excluded if they (1) involved individuals under 18 years of age; (2) used non-RCT designs such as cohort studies, case-control studies, or cross-sectional studies; (3) were review articles or conference abstracts; (4) lacked a control group; or (5) involved cadaveric or animal models.

### Systematic search for trials

We conducted a comprehensive search for relevant RCTs using the PubMed database of the National Library of Medicine, EMBASE, Web of Science, and Scopus. A combination of MeSH terms, platelet-rich plasma, leukocyte-rich, hyaluronic acid, LR-PRP, HA, and knee osteoarthritis, was used to maximize sensitivity. We also manually searched the reference lists from all reviewed articles and relevant systematic reviews to identify additional eligible studies. RCTs included in our analysis were published between November 1, 2011, and July 14, 2023. Our comprehensive search did not identify any relevant RCTs published before November 1, 2011. A detailed search strategy, including all keywords and search terms, is available in supplemental appendix S1 (available online only at http://www.archives-pmr.org/). No RCTs published after this period are relevant to the objectives of our research. Two independent reviewers extracted data using an identical standardized approach. Full-text articles were assessed individually to determine eligibility, and any discrepancies were resolved through discussion until consensus was achieved.

The authors independently reviewed and extracted essential information from each study, such as the first author’s name, country of origin, publication year, study design, participant count, age and sex of patients, outcome measures, and follow-up duration. Data were collected from the text, tables, or graphs presented in the included articles.

### Outcome measurements

The outcome measures comprised (1) pain scores on the VAS and Numeric Rating Scale, both assessed on an 11-point scale (ranging from 0 to 10). For studies that used a 100-point scale, the original score was converted to an 11-point scale by dividing by 10. These two scales are commonly referred to as pain scores. Outcomes included: (1) pain scores; (2) scores on the WOMAC, including the total score as well as subscales for pain, physical function, and stiffness; and (3) adverse events.

### Quality assessment

The quality of evidence for all outcomes was assessed through the Grading of Recommendations, Assessment, Development, and Evaluation (GRADE) framework.^25^ This evaluation is composed of 5 key factors: risk of bias, inconsistency, indirectness, imprecision, and other potential biases. Based on the potential impact of additional research on the confidence in the estimated effects, the evidence was categorized into 1 of 4 levels: high, moderate, low, or very low certainty. Furthermore, the methodological quality of each study was assessed using the clinical relevance scale developed by the National Heart, Lung, and Blood Institute (NHLBI).^26^ The risk of bias for each included trial was independently evaluated by 2 authors using the Cochrane Risk of Bias Tool. This tool examines several bias-related domains, including random sequence generation, allocation concealment, blinding of participants and outcome assessors, incomplete outcome data, selective reporting, and other possible biases. Each domain was classified as having a low, high, or unclear risk of bias.

### Data analysis

The systematic review and meta-analysis were performed using Review Manager version 5.4 (Nordic Cochrane Centre, Cochrane Collaboration).^a^ Mean differences (MDs) and SDs were used to compare continuous outcomes. All results were presented with 95% CIs, and a *P* value of ˂.05 was considered statistically significant. Heterogeneity among individual RCTs was assessed using the Higgins *I*² statistic. A fixed-effects model was applied when heterogeneity was low (*I*²<50%), whereas a random-effects model was used in cases of substantial heterogeneity (*I*²>50% and *P*<.10).

## Results

### Literature search

**Fig 1:**
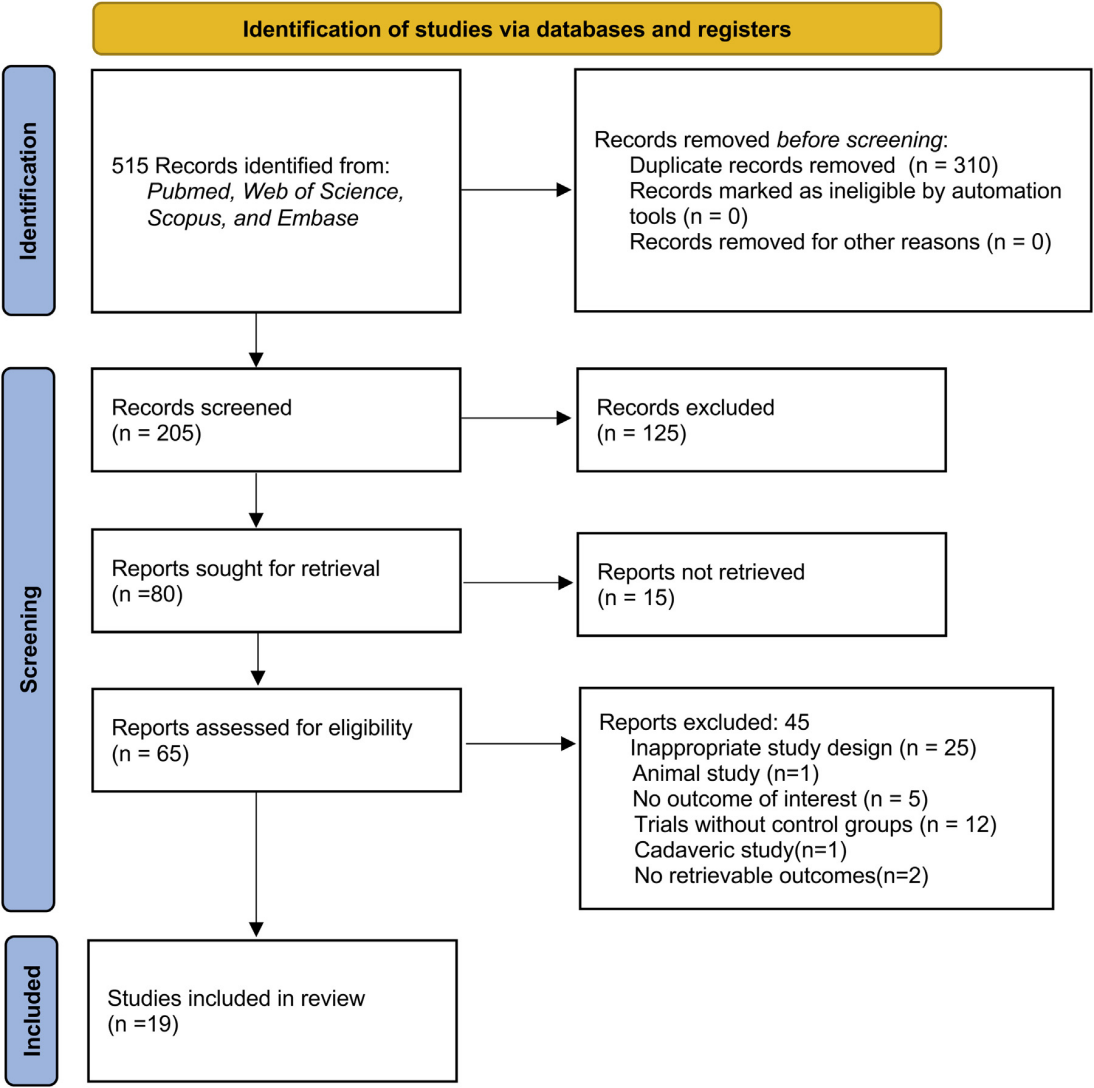
Preferred Reporting Items for Systematic Reviews and Meta-Analyses flow chart of literature search and selection process.

Figure 1 outlines the process of literature retrieval. The initial search across PubMed, EMBASE, Web of Science, and Scopus identified 515 potentially relevant studies. After removing 310 duplicates, 2 independent authors screened the remaining 205 studies based on their titles and abstracts. During this process, 125 studies were excluded as they did not meet the inclusion criteria. Full-text retrieval was attempted for the remaining 80 studies, but 15 were not retrieved as they were not provided in English. Eligibility assessment of the remaining 65 studies resulted in the exclusion of 45 because of reasons such as inappropriate study design, animal or cadaveric study, no outcome of interest, trials without control groups, or no retrievable outcomes.

### Main characteristics of included trials

The main characteristics of the included RCTs are summarized in table 1. A total of 19 RCTs were deemed eligible to be included in this meta-analysis, involving 1771 patients. Of these, 878 patients received LR-PRP injections, whereas 893 patients received HA injections. The studies were published between 2011 and 2023. Five studies were conducted in Italy,^19^^,^^20^^,^^27^^,^^28^^,^^37^ 5 in Turkey,^31^^,^^33^^,^^39^^,^^40^^,^^43^ 2 in China,^35^^,^^36^ 1 in each of the following countries: Slovakia,^29^ Egypt,^34^ South Korea,^41^ Brazil,^38^ Australia,^32^ Serbia,^42^ and Iran.^30^ Detailed characteristics and outcomes for the involved patients are provided in table 1, including study type, patient numbers, follow-up period, age, sex, knee OA grade (Kellgren-Lawrence classification), and outcome measurements.

**Table 1:** General characteristics of the included studies.

Included Trials	Groups	Patients (N)	Follow-up period (mo)	Age (y, LR-PRP/HA)	Sex (Male/Female, N)	Knee OA Grade (Kellgren-Lawrence Classification)	Outcome Measurements	Total Platelet Dose/Concentration Factor/Injection Volume
Kon et al^27^ (Italy)	LR-PRP HA	50 50	2, 6	50.6±13.8 54.9±12.6	30/20 25/25	0-4	EQ-VAS, IKDC, adverse events	NR/4.8x/5 mL
Filardo et al^28^ (Italy)	LR-PRP HA	54 55	2, 6, 12	55 58	37/17 31/24	1-3	IKDC	NR/5.4x/5 mL
Spaková et al^29^ (Slovakia)	LR-PRP HA	60 60	3, 6	52.80±12.43 53.20±14.53	33/27 31/29	1-3	WOMAC, adverse events	NR/∼3.0x/3 mL
Raeissadat et al^30^ (Iran)	LR-PRP HA	77 62	12	56.85±9.13 61.13±7.48	8/69 15/47	1-4	WOMAC	NR/3.2x/2 mL
Filardo et al^19^ (Italy)	LR-PRP HA	94 89	2, 6, 12	53.32±13.2 57.55±11.8	60/34 52/37	1-3	EQ-VAS, IKDC, adverse events	NR/5.4x/5 mL
Görmeli et al^31^ (Turkey)	LR-PRP HA	39 39	6	53.8±23.1 53.5±14	16/23 17/22	1-4	EQ-VAS, IKDC	NR/>4x/3 mL
Paterson et al^32^ (Australia)	LR-PRP HA	11 10	1, 3	49.91±13.72 52.70±10.30	8/3 7/3	2-3	VAS, adverse events	NR/2.2x/2.2 mL
Duymus et al^33^ (Turkey)	LR-PRP HA	33 34	1, 3, 6, 12	60.4±5.1 60.3±9.1	1/32 1/33	2-3	WOMAC, VAS	NR/5.9x/3 mL
Ahmad et al^34^ (Egypt)	LR-PRP HA	45 44	3, 6	56.2±6.8 56.8±7.4	14/31 14/30	1-3	VAS, IKDC	NR/2.9x/3 mL
Yu et al^35^ (China)	LR-PRP HA	104 88	12	46.2±8.6 51.5±9.3	50/54 48/40	1-4	WOMAC, adverse events	NR/5.0x/4 mL
Su et al^36^ (China)	LR-PRP HA	25 30	1, 3, 6, 12, 18	54.16±6.56 53.13±6.41	11/14 12/18	2-3	WOMAC, VAS, adverse events	NR/4.7x/4 mL
Tavassoli et al^37^ (Italy)	LR-PRP HA	28 27	1, 2, 3	66.04±7.58 63.30±8.87	6/22 8/19	1-2 (Ahlbäck classification)	WOMAC, VAS, adverse events	NR/3.6x/5 mL
Di Martino et al^20^ (Italy)	LR-PRP HA	85 82	2, 6, 12, 24	52.7±13.2 57.5±11.7	53/32 47/35	1-3	EQ-VAS, IKDC, adverse events	NR/5.4x/5 mL
Arliani et al^38^ (Brazil)	LR-PRP HA	14 15	1,3,6	62.78±6.10 63.40±4.99	3/11 2/13	2-3	WOMAC, VAS, adverse events	NR/∼5.0x/5 mL
Yaradilmis et al^39^ (Turkey)	LR-PRP HA	30 30	2,6,12	60.3±7.65 63±9.17	4/26 4/26	2-3	WOMAC, VAS, adverse events	NR/5.4x/3 mL
Kesiktas et al^40^ (Turkey)	LR-PRP HA	18 18	1,3	52. 7±8.3 55.1±10.3	16/2 14/4	2-4	WOMAC, VAS	NR/∼4.0x/4 mL
Park et al^41^ (South Korea)	LR-PRP HA	55 55	1.5, 3, 6	60.6±8.2 62.3±9.6	16/39 8/47	1-3	WOMAC, VAS, adverse events	NR/3.9x/5 mL
Dulic et al^42^ (Serbia)	LR-PRP HA	37 35	1,3,6,9,12	58.8±11.2 59.4±14.0	15/19 13/17	2-4	WOMAC, VAS, IKDC adverse events	NR/3.5x/3 mL
Küçükakkaş et al^43^ (Turkey)	LR-PRP HA	20 20	1,6	57.5±10.6 57.0±10.1	4/16 6/14	2-3	WOMAC, VAS	NR/4.0x/3 mL

Abbreviations: EQ-VAS, EuroQol Visual Analog Scale; IKDC, International Knee Documentation Committee; N, numbers; NR, not reported; NRS, Numeric Rating Scale.

### Quality assessment of the studies

The quality of the included studies was evaluated using the NHLBI clinical relevance scale (table 2). Based on the NHLBI quality assessment, the included RCTs were evaluated by 5 factors: patient, interventions, outcomes, effect size, and benefit versus harm. As for the grading, thirteen studies^19^^,^^20^^,^^27^^,^^29^, ^30^, ^31^^,^^33^^,^^37^, ^38^, ^39^^,^^41^^,^^42^ received a score of 5 of 5, four^28^^,^^35^^,^^38^^,^^40^ received a score of 4 of 5, and two^32^^,^^34^ received a score of 3 of 5.

**Table 2:** The clinical relevance grade of the included studies (NHLBI quality assessment).

Included Trials	Y	Patient	Interventions	Outcomes	Effect Size	Benefit vs Harm	Grade
Kon et al^27^ (Italy)	2011	+	+	+	+	+	5/5
Filardo et al^28^ (Italy)	2012	+	+	+	+	+	4/5
Spaková et al^29^ (Slovakia)	2012	+	+	+	+	+	5/5
Raeissadat et al^30^ (Iran)	2015	+	+	+	+	+	5/5
Filardo et al^19^ (Italy)	2015	+	+	+	-	+	5/5
Görmeli et al^31^ (Turkey)	2015	+	+	+	+	+	5/5
Paterson et al^32^ (Australia)	2016	-	+	+	-	+	3/5
Duymus et al^33^ (Turkey)	2017	+	+	+	+	+	5/5
Ahmad et al^34^ (Egypt)	2018	+	+	-	-	+	3/5
Yu et al^35^ (China)	2018	+	+	+	+	-	4/5
Su et al^36^ (China)	2018	+	+	+	+	+	5/5
Tavassoli et al^37^ (Italy)	2019	+	+	+	+	+	5/5
Di Martino et al^20^ (Italy)	2019	+	+	+	+	+	5/5
Arliani et al^38^ (Brazil)	2020	+	+	+	-	+	4/5
Yaradilmis et al^39^ (Turkey)	2020	+	+	+	+	+	5/5
Kesiktas et al^40^ (Turkey)	2020	+	+	+	-	+	4/5
Park et al^41^ (South Korea)	2021	+	+	+	+	+	5/5
Dulic et al^42^ (Serbia)	2021	+	+	+	+	+	5/5
Küçükakkaş et al^43^ (Turkey)	2022	+	+	+	+	+	5/5

### GRADE evidence evaluation

The GRADE evidence evaluation was used to evaluate the quality of the evidence for the primary, secondary, and tertiary outcomes, including pain scores, total WOMAC scores, WOMAC pain scores, WOMAC physical function scores, and WOMAC stiffness scores (table 3). The overall quality of evidence was generally high to moderate. Although all included studies were RCTs, some lacked details regarding allocation concealment and blinding of outcome assessment. In addition, some visual inconsistencies were found on the forest plots and showed moderate heterogeneity (*I*^2^>50%), which may have further influenced the reliability of the findings.

**Table 3:** The GRADE evidence evaluation.

Outcomes	Illustrative Comparative Risks (95% CI)	No. of Participants (Studies)	Certainty of the Evidence (GRADE)	Comments
Pain score at 1 mo	The mean meta-analysis of pain score at 1 mo in the LR-PRP groups was 0.12 SDs lower (0.48 lower to 0.23 higher)	219 (5 RCTs)	⨁⨁⨁⨁ High*	SMD −0.12 (−0.48 to 0.23)
Pain score at 3 mo	The mean meta-analysis of pain score at 3 mo in the LR-PRP groups was 0.11 SDs lower (0.16 lower to 0.39 higher)	380 (6 RCTs)	⨁⨁⨁⨁ High*	SMD −0.11 (−0.16 to 0.39)
Pain score at 6 mo	The mean meta-analysis of pain score at 6 mo in the LR-PRP groups was 0.14 SDs lower (0.56 lower to 0.84 higher)	963 (10 RCTs)	⨁⨁⨁⊝ Moderate*^,^†	SMD −0.14 (−0.56 to 0.84)
Pain score at 12 mo	The mean meta-analysis of pain score at 12 mo in the LR-PRP groups was 0.14 SDs lower (0.56 lower to 0.84 higher)	532 (5 RCTs)	⨁⨁⨁⊝ Moderate*^,^†	SMD −0.14 (−0.56 to 0.84)
Total WOMAC score at 1 mo	The mean meta-analysis of total WOMAC score at 1 mo in the LR-PRP groups was 2.65 SDs lower (6.75 lower to 1.45 higher)	303 (6 RCTs)	⨁⨁⨁⨁ High*	SMD −2.65 (−6.75 to 1.45)
Total WOMAC score at 3 mo	The mean meta-analysis of total WOMAC score at 3 mo in the LR-PRP groups was 2.9 SDs lower (7.14 lower to 1.34 higher)	493 (7 RCTs)	⨁⨁⨁⊝ Moderate*^,^†	SMD −2.9 (−7.14 to 1.34)
Total WOMAC score at 6 mo	The mean meta-analysis of total WOMAC score at 6 mo in the LR-PRP groups was 4.45 SDs lower (9.81 lower to 0.92 higher)	553 (8 RCTs)	⨁⨁⨁⊝ Moderate*^,^†	SMD −4.45 (−9.81 to 0.92)
Total WOMAC score at 12 mo	The mean meta-analysis of total WOMAC score at 12 mo in the LR-PRP groups was 7.44 SDs lower (17.52 lower to 2.64 higher)	393 (5 RCTs)	⨁⨁⨁⊝ Moderate*^,^†	SMD −7.44 (−17.52 to 2.64)
WOMAC pain score at 1 mo	The mean meta-analysis of WOMAC pain score at 1 mo in the LR-PRP groups was 0.18 SDs lower (1.17 lower to 1.52 higher)	158 (3 RCTs)	⨁⨁⨁⨁ High*	SMD −0.18 (−1.17 to 1.52)
WOMAC pain score at 3 mo	The mean meta-analysis of WOMAC pain score at 3 mo in the LR-PRP groups was 0.51 SDs lower (2.61 lower to 1.59 higher)	268 (4 RCTs)	⨁⨁⨁⊝ Moderate*^,^†	SMD −0.51 (−2.61 to 1.59)
WOMAC pain score at 6 mo	The mean meta-analysis of WOMAC pain score at 6 mo in the LR-PRP groups was 1.62 SDs lower (5.95 lower to 2.71 higher)	292 (4 RCTs)	⨁⨁⨁⊝ Moderate*^,^†	SMD −1.62 (−5.95 to 2.71)
WOMAC pain score at 12 mo	The mean meta-analysis of WOMAC pain score at 12 mo in the LR-PRP groups was 3.61 SDs lower (9.08 lower to 1.85 higher)	376 (4 RCTs)	⨁⨁⨁⊝ Moderate*^,^†	SMD −3.61 (−9.08 to 1.85)
WOMAC physical function score at 1 mo	The mean meta-analysis of WOMAC physical function score at 1 mo in the LR-PRP groups was 1.82 SDs lower (6.26 lower to 2.62 higher)	182 (3 RCTs)	⨁⨁⨁⨁ High*	SMD −1.82 (−6.26 to 2.62)
WOMAC physical function score at 3 mo	The mean meta-analysis of WOMAC physical function score at 3 mo in the LR-PRP groups was 0.57 SDs lower (4.37 lower to 3.24 higher)	292 (4 RCTs)	⨁⨁⨁⊝ Moderate*^,^†	SMD −0.57 (−4.37 to 3.24)
WOMAC physical function score at 6 mo	The mean meta-analysis of WOMAC physical function score at 6 mo in the LR-PRP groups was 0.16 SDs lower (6.51 lower to 6.19 higher)	292 (4 RCTs)	⨁⨁⨁⊝ Moderate*^,^†	SMD −0.16 (−6.51 to 6.19)
WOMAC physical function score at 12 mo	The mean meta-analysis of WOMAC physical function score at 12 mo in the LR-PRP groups was 6.37 SDs lower (12.51 lower to 0.24 lower)	321 (4 RCTs)	⨁⨁⨁⊝ Moderate*^,^†	SMD −6.37 (−12.51 to −0.24)
WOMAC stiffness score at 1 mo	The mean meta-analysis of WOMAC stiffness score at 1 mo in the LR-PRP groups was 0.12 SDs lower (0.66 lower to 0.41 higher)	158 (3 RCTs)	⨁⨁⨁⨁ High*	SMD −0.12 (−0.66 to 0.41)
WOMAC stiffness score at 3 mo	The mean meta-analysis of WOMAC stiffness score at 3 mo in the LR-PRP groups was 0.06 SDs lower (0.71 lower to 0.58 higher)	268 (4 RCTs)	⨁⨁⨁⊝ Moderate*^,^†	SMD −0.06 (−0.71 to 0.58)
WOMAC stiffness score at 6 mo	The mean meta-analysis of WOMAC stiffness score at 6 mo in the LR-PRP groups was 0.21 SDs lower (0.7 lower to 0.27 higher)	292 (4 RCTs)	⨁⨁⨁⊝ Moderate*^,^†	SMD −0.21 (−0.7 to 0.27)
WOMAC stiffness score at 12 mo	The mean meta-analysis of WOMAC stiffness score at 12 mo in the LR-PRP groups was 0.63 SDs lower (0.98 lower to 0.27 lower)	321 (4 RCTs)	⨁⨁⨁⨁ High*	SMD −0.63 (−0.98 to −0.27)

NOTE. High certainty: we are very confident that the true effect lies close to that of the estimated effect. Moderate certainty: we are moderately confident in the effect estimate that the true effect is likely to be close to the estimate of the effect, but there is a possibility that it is substantially different. Low certainty: our confidence in the effect estimate is limited and the true effect may be substantially different from the estimate of the effect. Very low certainty: we have very little confidence in the effect estimate and the true effect is likely to be substantially different from the estimate of effect.

Abbreviation: SMD, standardized mean difference.

⁎ Some concerns in allocation concealment and blinding of outcome assessment, but not rated down for risk of bias.

† Visual inconsistency and statistical analyses also show heterogeneity.

### Risk of bias assessment

**Fig 2:**
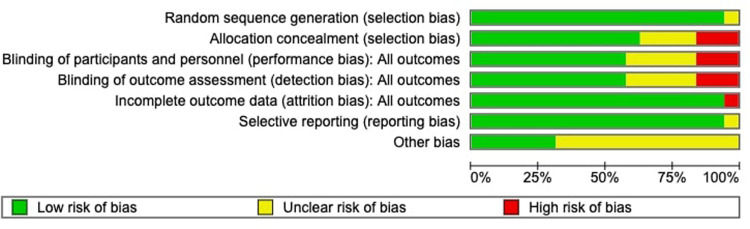
Risk of bias graph.

**Fig 3:**
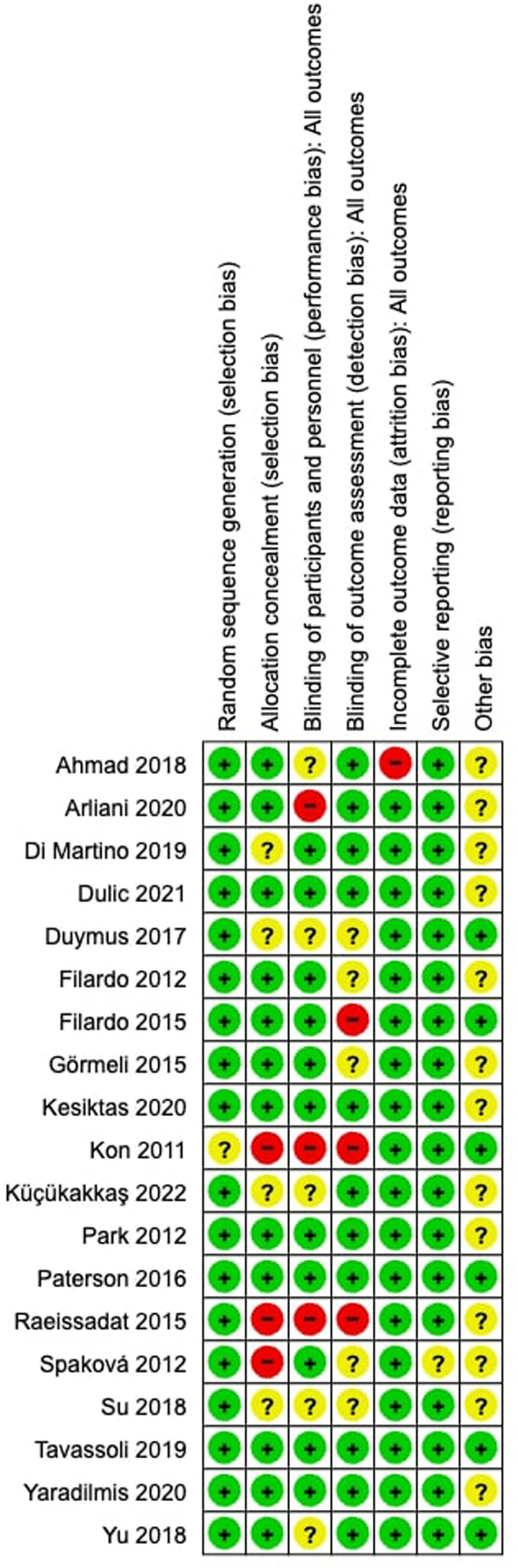
Risk of bias summary.

Fig 2, Fig 3 summarize the risk of bias among the included trials. Adequate random sequence generation was achieved in all 8 studies except for 1 study by Kon et al,^27^ whereas allocation concealment was explicitly reported in 13 studies.^19^^,^^28^^,^^31^^,^^32^^,^^34^^,^^35^^,^^37^, ^38^, ^39^, ^40^, ^41^, ^42^ Blinding of participants and personnel was implemented in 11 studies^19^^,^^20^^,^^28^^,^^29^^,^^31^^,^^32^^,^^37^^,^^39^^,^^40^^,^^41^^,^^42^ and blinding of outcome assessment was also documented in eleven studies.^20^^,^^32^^,^^34^^,^^35^^,^^37^, ^38^, ^39^, ^40^, ^41^, ^42^, ^43^ Seven studies^20^^,^^32^^,^^37^^,^^39^^,^^40^^,^^41^^,^^42^ were classified as double-blinded.^44^, ^45^, ^46^, ^47^ One of the included studies^34^ showed evidence of incomplete outcome data. One study presented an unclear risk of selective reporting,^29^ and 13 studies demonstrated an unclear risk of other potential biases.^20^^,^^28^, ^29^, ^30^, ^31^^,^^34^^,^^36^^,^^38^, ^39^, ^40^, ^41^, ^42^, ^43^

### Meta-analyses primary outcome: pain scores (1, 3, 6, and 12 months posttreatment)

**Fig 4:**
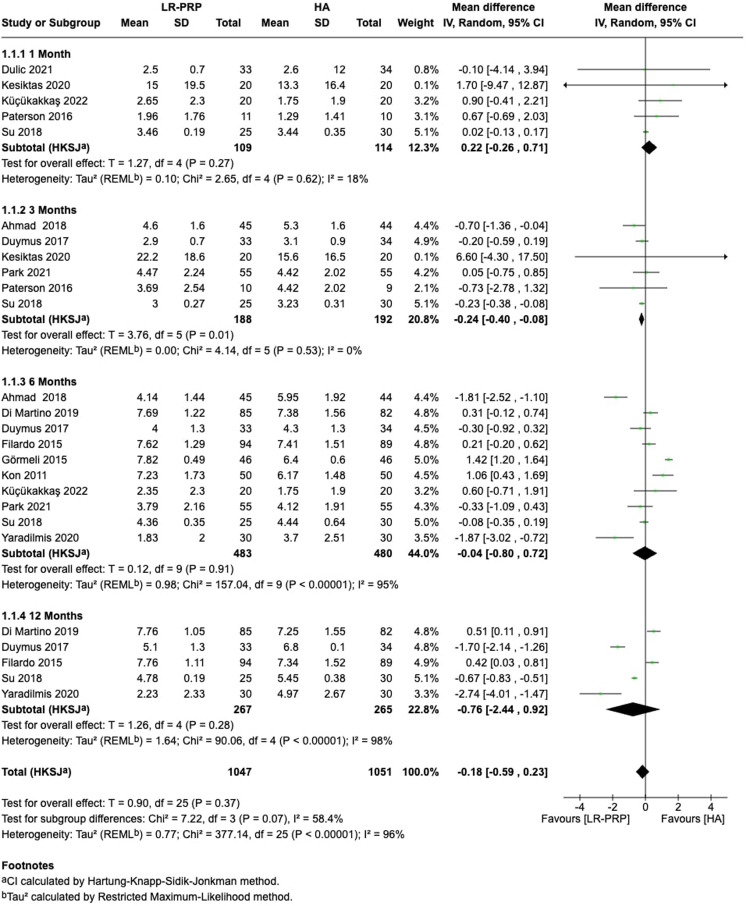
LR-PRP versus HA: Meta-analysis of Pain Scores at 1, 3, 6, 12 months. Abbreviation: IV, inverse variance.

Figure 4 compares LR-PRP and HA injections based on pain scores assessed using either the VAS or the Numeric Rating Scale. A random-effects model was employed to account for the substantial heterogeneity observed across the included studies (*I*²=96%; *P*<.0001). Subgroup analysis according to the follow-up periods after treatment was performed.

At the 1-month follow-up, 5 studies reported outcomes, with minimal heterogeneity (*I*²=18%), and the difference between the 2 treatment groups was not statistically significant (MD=0.22; 95% CI, −0.26 to 0.71; *P*=0.27). At the 3-month follow-up, 6 studies reported outcomes with minimal heterogeneity (*I*²=0%). A statistically significant difference was observed between the 2 treatment groups (MD=−0.24; 95% CI, −0.40 to −0.08; *P*=.01), indicating that the LR-PRP group was superior to the HA group. At the 6-month follow-up, 10 studies reported outcomes, with moderate heterogeneity (*I*²=95%), and results indicated comparable efficacy between LR-PRP and HA groups (MD=−0.04; 95% CI, −0.80 to 0.72; *P*=.91). At the 12-month follow-up, 5 studies reported outcomes, with moderate heterogeneity (*I*²=98%), and no significant long-term benefit was detected for either group (MD=−0.18; 95% CI, −0.59 to 0.23; *P*=.37).

Subgroup analysis revealed a significant difference in pain scores between the LR-PRP and HA groups at 3 months posttreatment, whereas no significant differences were found at 1, 6, or 12 months.

### Meta-analyses secondary outcomes: WOMAC total scores (1, 3, 6, and 12 months posttreatment)

**Fig 5:**
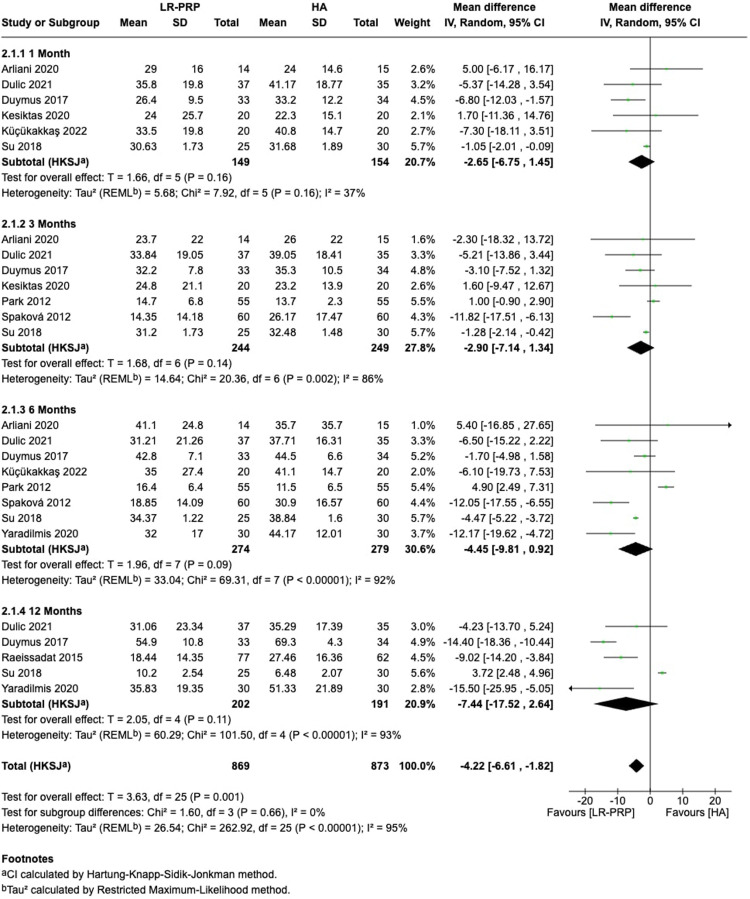
LR-PRP versus HA: Meta-analysis of total WOMAC Scores at 1, 3, 6, 12 months. Abbreviation: IV, inverse variance.

Figure 5 compares LR-PRP and HA injections based on total WOMAC scores. We further performed subgroup analysis according to the follow-up periods after treatment. At the 1-month follow-up, 6 studies reported outcomes, with mild heterogeneity (*I*²=37%), and no statistically significant difference was observed between the 2 treatment groups (MD=−2.65; 95% CI, −6.75 to 1.45; *P*=.16). At the 3-month follow-up, 7 studies reported outcomes, with moderate heterogeneity (*I*²=86%), and statistical analysis revealed similar outcomes for both groups (MD=−2.90; 95% CI, −7.14 to 1.34; *P*=.14). At the 6-month follow-up, 8 studies reported outcomes, with moderate heterogeneity (*I*²=92%), and the results indicated comparable efficacy between the 2 treatment groups (MD=−4.45; 95% CI, −9.81 to 0.92; *P*=.09). At the 12-month follow-up, 5 studies reported outcomes, with moderate heterogeneity (*I*²=93%), and there was no significant long-term benefit observed between the 2 treatment groups (MD=−7.44; 95% CI, −17.52 to 2.64; *P*=.11).

Subgroup analysis revealed that the differences in total WOMAC scores between LR-PRP and HA injections were not statistically significant at 1, 3, 6, and 12 months after treatment.

### Meta-analyses tertiary outcomes: WOMAC pain, stiffness, and physical function scores (1, 3, 6, and 12 months posttreatment)

**WOMAC pain scores**

**Fig 6:**
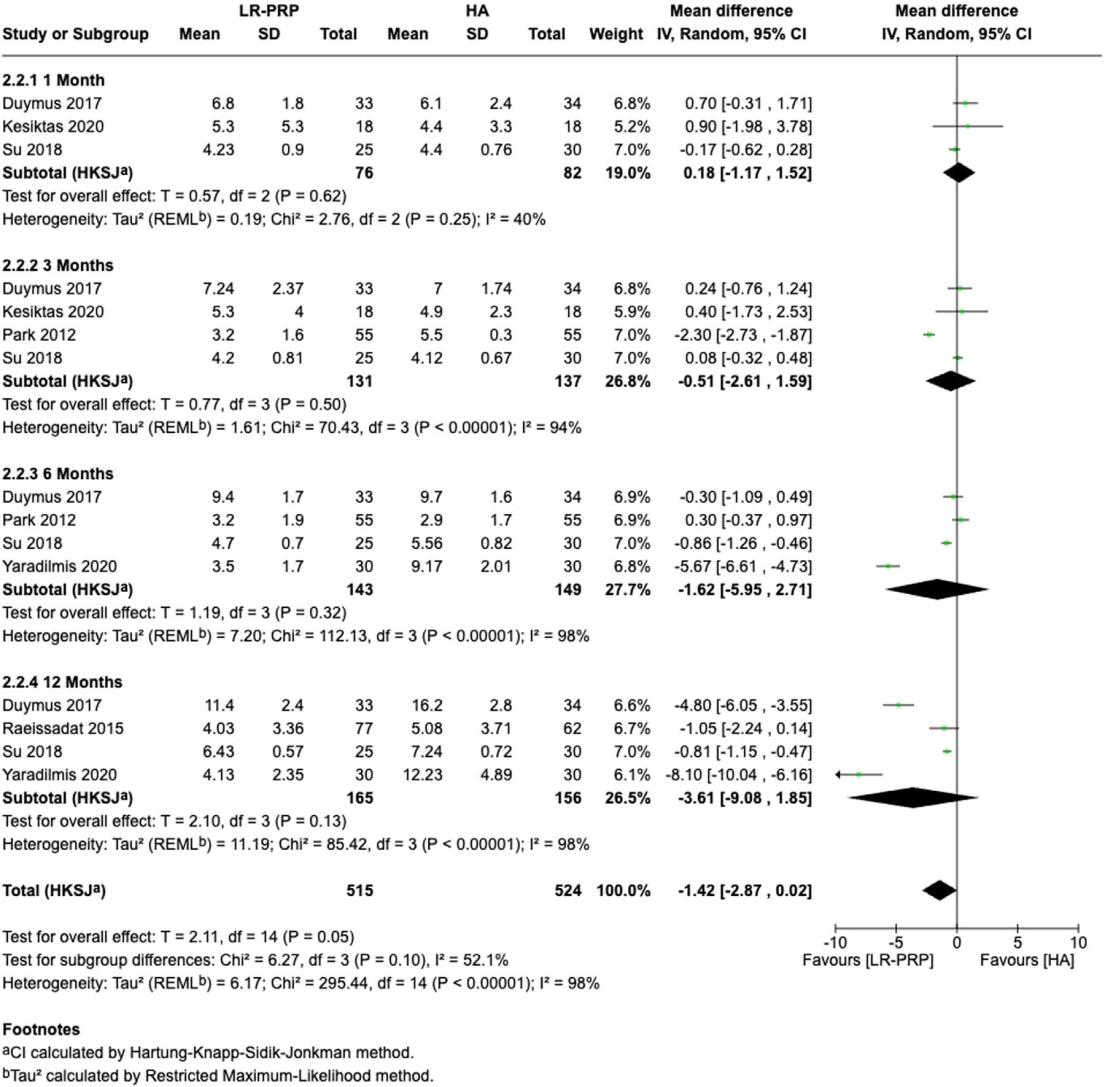
LR-PRP versus HA: Meta-analysis of WOMAC Pain Scores at 1, 3, 6, 12 months. Abbreviation: IV, inverse variance.

Figure 6 compares LR-PRP and HA injections based on WOMAC pain scores. We further performed subgroup analysis according to the follow-up periods after treatment. At the 1-month follow-up, 3 studies reported outcomes, with mild heterogeneity (*I*²=40%), and there was no statistically significant difference observed between the 2 treatment groups (MD=0.18; 95% CI, −1.17 to 1.52; *P*=.62). At the 3-month follow-up, 4 studies reported outcomes, with moderate heterogeneity (*I*²=94%), and there was no statistically significant difference observed between the 2 treatment groups (MD=−0.51; 95% CI, −2.61 to 1.59; *P*=.50). At the 6-month follow-up, 4 studies reported outcomes, with moderate heterogeneity (*I*²=98%), and there was no statistically significant difference observed between the 2 treatment groups (MD=−1.62; 95% CI, −5.95 to 2.71; *P*=.32). At the 12-month follow-up, 4 studies reported outcomes, with moderate heterogeneity (*I*²=98%), and there was no statistically significant difference observed between the 2 treatment groups (MD=−3.61; 95% CI, −9.08 to 1.85; *P*=.13).

Subgroup analysis revealed that the differences in WOMAC pain scores between LR-PRP and HA injections were not statistically significant at 1, 3, 6, and 12 months posttreatment.

### WOMAC physical function scores

**Fig 7:**
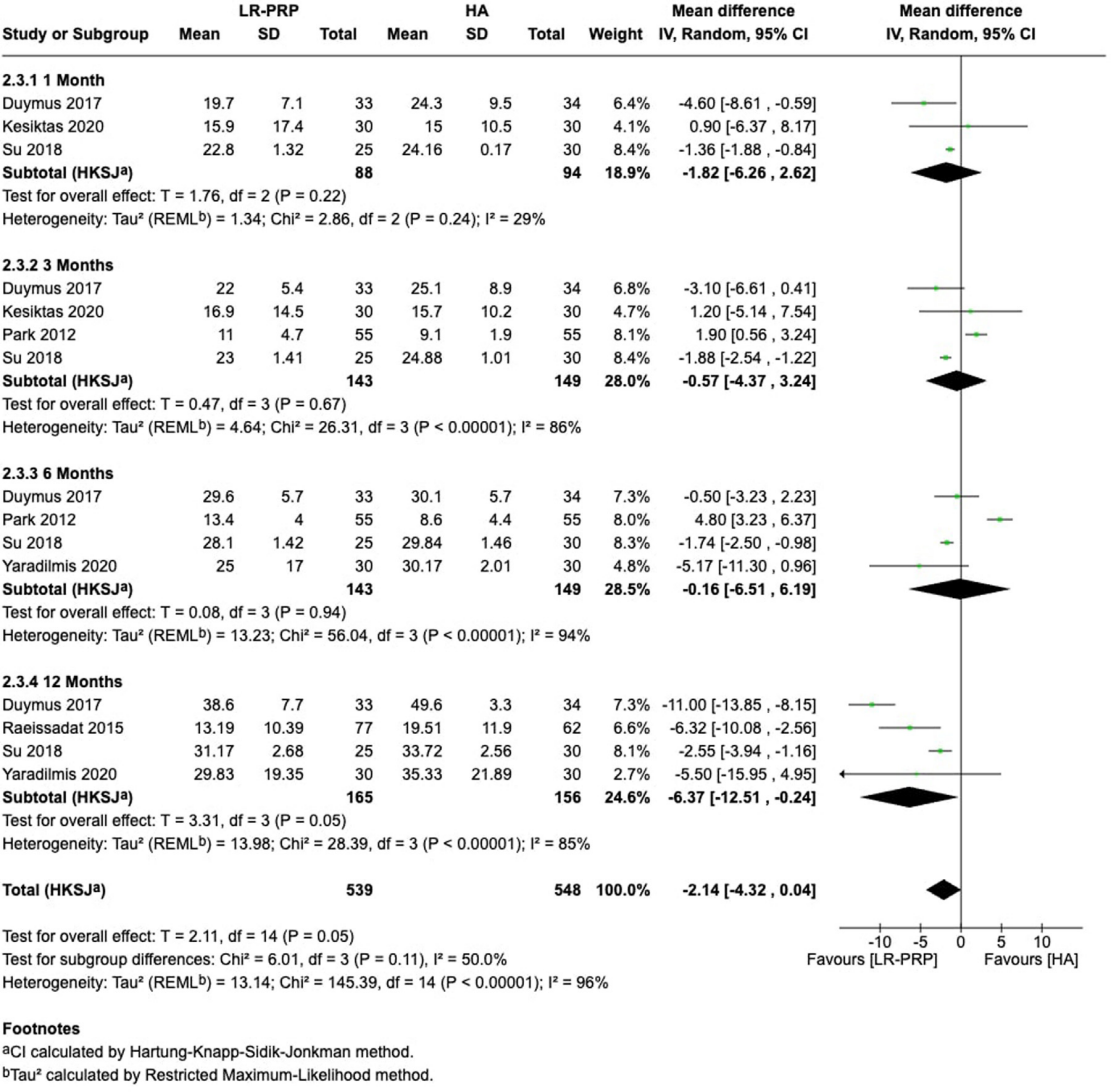
LR-PRP versus HA: Meta-analysis of WOMAC Physical Function Scores at 1, 3, 6, 12 months. Abbreviation: IV, inverse variance.

Figure 7 compares LR-PRP and HA injections based on WOMAC physical function scores. We further performed subgroup analysis according to the follow-up periods after treatment. At the 1-month follow-up, 3 studies reported outcomes, with mild heterogeneity (*I*²=29%), and there was no statistically significant difference observed between the 2 treatment groups (MD=−1.82; 95% CI, −6.26 to 2.62; *P*=.22). At the 3-month follow-up, 4 studies reported outcomes, with moderate heterogeneity (*I*²=86%), and there was no statistically significant difference observed between the 2 treatment groups (MD=−0.57; 95% CI, −4.37 to 3.24; *P*=.67). At the 6-month follow-up, 4 studies reported outcomes, with moderate heterogeneity (*I*²=94%), and there was no statistically significant difference observed between the 2 treatment groups (MD=−0.16; 95% CI, −6.51 to 6.19; *P*=.94). At the 12-month follow-up, 4 studies reported outcomes, with moderate heterogeneity (*I*²=85%), and found that LR-PRP was associated with a significant reduction in WOMAC physical function scores compared with the HA group (MD=−6.37; 95% CI, −12.51 to −0.24; *P*=.05).

Subgroup analysis revealed a statistically significant difference in WOMAC physical function scores between the LR-PRP and HA groups at 12 months posttreatment, whereas no significant differences were found at 1, 3, or 6 months.

### WOMAC stiffness scores

**Fig 8:**
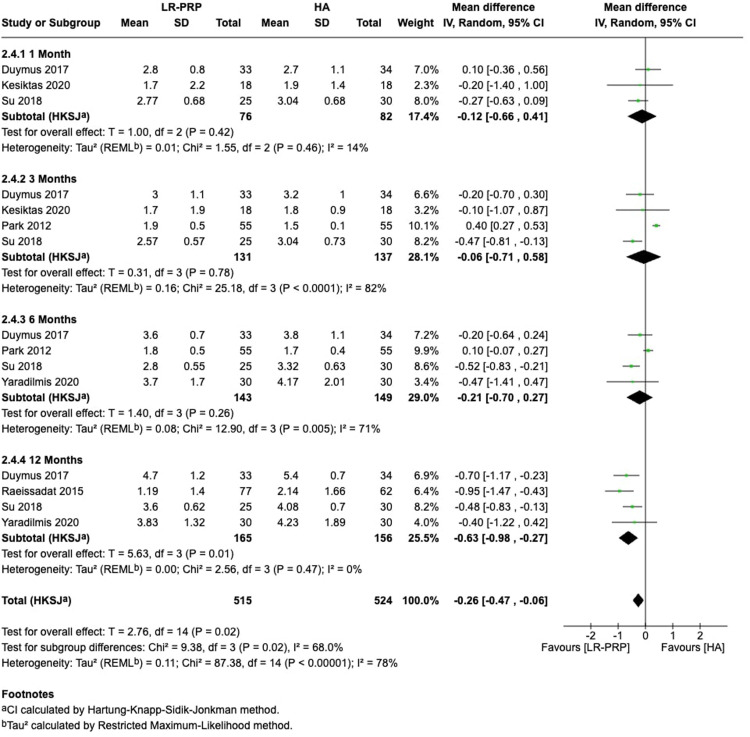
LR-PRP versus HA: Meta-analysis of WOMAC Stiffness scores at 1, 3, 6, 12 months. Abbreviation: IV, inverse variance.

Figure 8 compares LR-PRP and HA injections based on WOMAC stiffness scores. We further performed subgroup analysis according to the follow-up periods after treatment. At the 1-month follow-up, 3 studies reported outcomes, with mild heterogeneity (*I*²=14%), and there was no statistically significant difference observed between the 2 treatment groups (MD=−0.12; 95% CI, −0.66 to 0.41; *P*=.42). At the 3-month follow-up, 4 studies reported outcomes, with moderate heterogeneity (*I*²=82%) and no statistically significant difference between the 2 treatments (MD=−0.06; 95% CI, −0.71 to 0.58; *P*=.78). At the 6-month follow-up, 4 studies reported outcomes, with moderate heterogeneity (*I*²=71%), and there was no statistically significant difference observed between the 2 treatment groups (MD=−0.21; 95% CI, −0.70 to 0.27; *P*=.26). At the 12-month follow-up, 4 studies reported outcomes, with minimal heterogeneity (*I*²=0%), and found that LR-PRP was associated with a significant reduction in WOMAC stiffness scores compared with the HA group (MD=−0.63; 95% CI, −0.98 to −0.27; *P*=.01). The subgroup analysis demonstrated a statistically significant difference in WOMAC stiffness scores between the LR-PRP and HA groups at 12 months after treatment; however, no significant differences were observed at 1, 3, or 6 months.

### Adverse events

**Fig 9:**
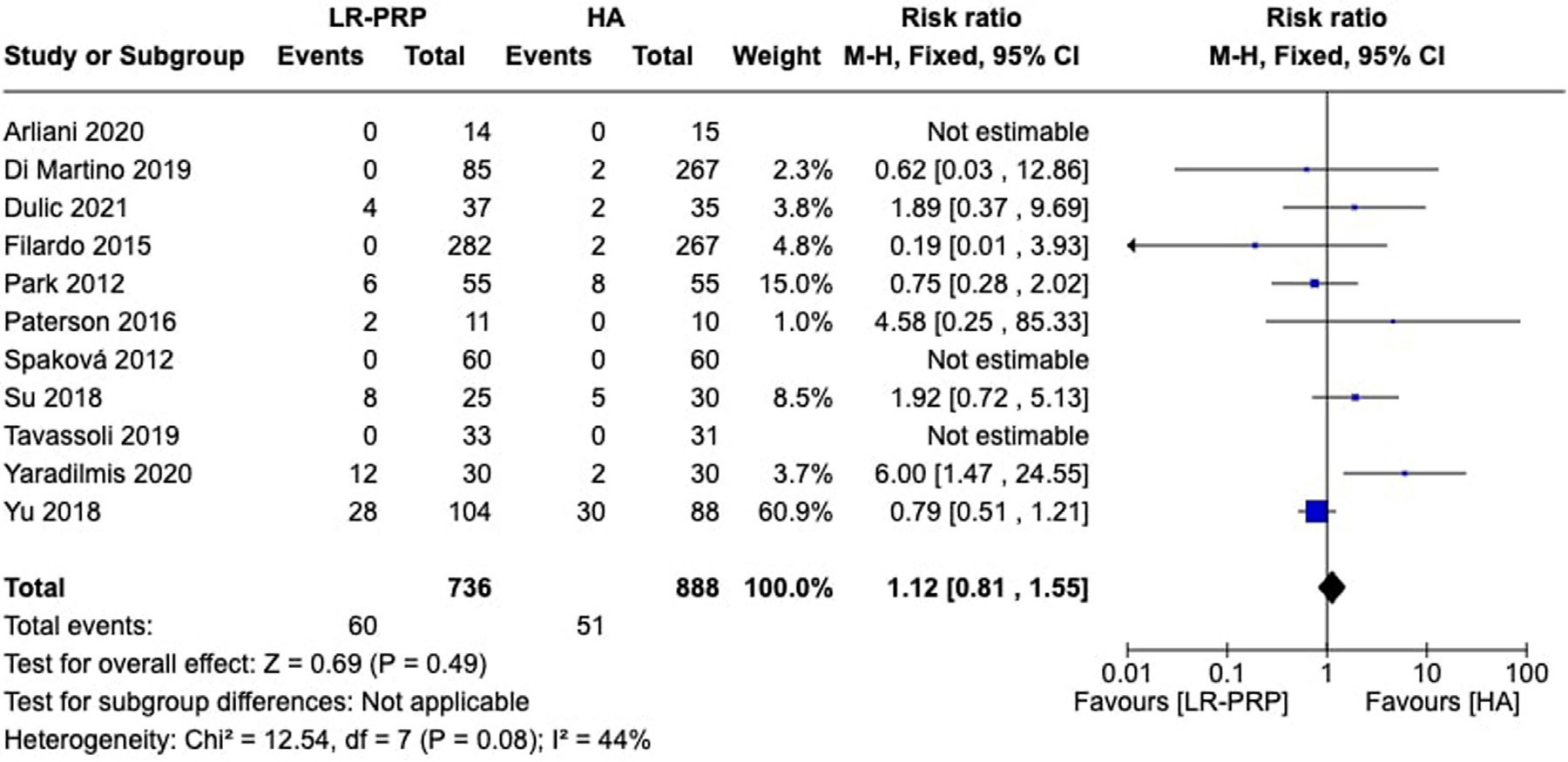
LR-PRP versus HA: Meta-analysis of adverse effects. Abbreviation: M-H, Mantel Haenszel.

Figure 9 compares the adverse events between the LR-PRP and HA groups for knee OA, incorporating data from 11 RCTs.^19^^,^^20^^,^^29^^,^^32^^,^^35^, ^36^, ^37^, ^38^, ^39^^,^^41^^,^^42^ The most reported adverse events were increased lower limb swelling, lower limb pain, nausea, vomiting, and fatigue. No severe adverse events were reported in either group. The results indicated no significant difference between LR-PRP and HA (RR=1.12; 95% CI, 0.81-1.55; *P*=.49), suggesting that both treatments exhibit similar safety clinical profiles.

## Discussion

In our meta-analysis, we found that intra-articular injection of LR-PRP in patients with knee OA only results in significant pain relief at 3 months posttreatment, but no long-term pain relief. However, it does lead to functional improvements, as measured by the WOMAC physical function scores and WOMAC stiffness scores, at the 12-month follow-up, compared with HA injections. The temporal dissociation between early pain relief and late functional improvement warrants discussion. The significant pain reduction observed at 3 months may be attributed to the immediate anti-inflammatory modulation provided by the bioactive molecules in PRP.^48^ In contrast, functional improvements, such as physical function and stiffness, often require structural tissue adaptation and neuromuscular recovery, which are gradual processes. The sustained functional benefits observed at 12 months in the LR-PRP group suggest that the biological effects of platelet-derived growth factors may initiate a slower but more durable reparative cascade compared with the transient rheological effects of HA.^20^ Regarding adverse events, neither treatment showed any major adverse events, and the incidence of mild adverse events was not significantly different between the 2 groups.

In contrast to our results, a meta-analysis by Tan et al^49^ indicated that PRP may provide better pain relief and functional improvement than HA for a duration ranging from 3 to 12 months. Likewise, a meta-analysis by Dong et al^50^ demonstrated that intra-articular PRP injections yielded superior outcomes compared with HA in patients with knee OA, with significant improvements in pain scores, and WOMAC pain, stiffness, and physical function scores at follow-up intervals of 1, 2, 3, 6, and 12 months. However, neither of the 2 meta-analyses differentiated between PRP types nor did they specifically investigate the efficacy of LR-PRP compared with HA. Instead, they analyzed all PRP variants, encompassing both LR-PRP and LP-PRP, as a collective entity.

Although the meta-analysis showed a superior efficacy of LR-PRP compared with HA at 12 months posttreatment, as measured by the WOMAC physical function and stiffness scores, our study also found no significant differences between the LR-PRP and HA groups in WOMAC total scores, pain scores, physical function scores, and stiffness scores at 1, 3, and 6 months posttreatment. These findings are consistent with previous literature, as few studies have reported similar results regarding the lack of effect of LR-PRP between the 1- to 6-month period. A meta-analysis^51^ supports the superiority of PRP over HA, as evidenced by WOMAC total scores at 1, 6, and 12 months, and WOMAC physical function scores at 12 months. However, similarly, this study also did not separately examine the effects of LR-PRP. Another meta-analysis^52^ found that LR-PRP injection did not result in significantly greater pain relief compared with HA injection; nevertheless, LR-PRP showed superior outcomes in WOMAC total scores, pain scores, and physical function at 6 months posttreatment.

The superiority of the LR-PRP group over the HA group in WOMAC physical function and stiffness scores at the 12-month follow-up remains uncertain, because only a single injection was administered and the sustained effect of LR-PRP over 12 months may be limited. Several studies have raised concerns regarding the long-term efficacy of a single intra-articular LR-PRP injection for knee OA. Zhou et al^53^ demonstrated that LR-PRP improved WOMAC scores within 6 weeks; however, the effects diminished by the 12-month follow-up, with scores returning toward baseline levels. Similarly, a meta-analysis^49^ reported that although LR-PRP showed better outcomes than HA in WOMAC physical function and stiffness scores at 12 months, the evidence was inconclusive and marked by high heterogeneity. These findings prove that the benefit of a single LR-PRP injection may not be sustained over 12 months when assessed using WOMAC physical function and stiffness scores. The functional improvement may be attributed to various confounding factors, including patients’ lifestyle modifications, muscle strength training, rehabilitation programs, and the natural healing process of knee OA. Hannan et al^54^ found that 20%-30% of the patients with knee OA reported reduced pain and improved function over 1-2 years, despite no radiographic improvement, suggesting a natural healing process.

The role of leukocyte concentration in PRP for the injection treatment of knee OA remains controversial, with studies showing inconsistent conclusions regarding its impact on treatment outcomes. Some research suggests that LR-PRP may enhance inflammatory responses, leading to improved healing and tissue regeneration in certain conditions.^20^ Conversely, other studies have indicated that high leukocyte concentrations could exacerbate inflammation and potentially hinder tissue repair.^11^ For example, a study^55^ found that LP-PRP resulted in more favorable clinical outcomes in musculoskeletal injuries, whereas high leukocyte content in LR-PRP showed a less predictable effect. The variation in these results could be attributed to differences in preparation protocols, patient characteristics, and clinical indications. Moreover, several studies emphasize the importance of optimizing leukocyte concentrations for specific applications, as the inflammatory response induced by leukocytes might be beneficial in some cases but detrimental in others.^13^ Thus, further research is needed to establish standardized guidelines for leukocyte concentration in PRP preparations to maximize therapeutic efficacy across diverse clinical settings.

Another critical factor influencing clinical outcomes is the absolute dosage of platelets delivered. Although a specific subgroup analysis based on platelet dose was limited by the reporting variability across included studies, evidence suggests a dose-response relationship. High-dose PRP strategies may theoretically provide a more robust growth factor stimulus necessary for cartilage homeostasis in osteoarthritic knees. Conversely, variations in injectate volume and concentration factors among the analyzed trials likely contributed to the observed heterogeneity. Future standardization of reporting, specifically total platelet count per injection, is essential to define the optimal therapeutic threshold.

### Study limitations

First, although our systematic literature search concluded in July 2023, we acknowledge a recently published RCT by Romandini et al.^56^ This recent finding aligns with our observations regarding the clinical profile of LR-PRP and suggests that the exclusion of this trial because of the search date cutoff is unlikely to alter the overall conclusions of our meta-analysis. Second, significant heterogeneity across the included studies remained a major challenge. The substantial outcome heterogeneity (*I*² values) observed may be attributed to differences in study designs, including variations in LR-PRP protocols, follow-up duration, and outcome assessment methods. Specifically, the variability in centrifugation speeds, activation methods, and resulting leukocyte/platelet concentrations across different commercial kits represents an inherent limitation in the PRP literature. Although we strictly included only LR-PRP studies to minimize this, the lack of standardized characterization in some primary studies limits the comparability of the exact biological product administered. In addition, patient demographics, such as age, sex, body mass index, nationality, baseline pain severity, and knee OA grade, likely contributed to the observed heterogeneity. Previous research suggests that older patients or those with more advanced OA may exhibit different responses to PRP injections compared with younger individuals with less severe disease.^57^^,^^58^ Aging is associated with cellular senescence and a decline in the proliferative capacity of mesenchymal stem cells. Consequently, the autologous PRP derived from older patients may contain lower concentrations of functional growth factors and a reduced responsiveness of target chondrocytes, potentially diminishing the therapeutic efficacy compared with younger cohorts.^59^ Lastly, several of the included studies had relatively small sample sizes, which can result in overestimating the actual effect size in meta-analyses. Meta-analyses that involve a large proportion of small studies should be interpreted with caution, as the pooled estimates may be biased because of the inflated effect sizes.^60^

## Conclusions

In conclusion, our meta-analysis suggests that LR-PRP offers long-term functional improvements for patients with chronic knee OA, but no substantial evidence supports its effectiveness for short-term functional benefits. Intra-articular LR-PRP does not demonstrate long-term advantage in pain relief compared with intra-articular HA injections for patients with knee OA. Furthermore, the significant heterogeneity in patient demographics, LR-PRP protocols, and outcome measures across studies presents substantial challenges in drawing definitive conclusions. To improve the clinical applicability of LR-PRP, future research should focus on standardized protocols, larger sample sizes, and longer follow-up periods.

## Supplier

a. Review Manager 5.4; Cochrane Collaboration.

## Appendix Supplementary materials

Image, application 1

## Footnotes

Supplementary material associated with this article can be found, in the online version, at doi:10.1016/j.arrct.2025.100573.
