# Supplementary material for: Comparative Clinical Effectiveness of Intra-Articular Leukocyte-Rich Platelet-Rich Plasma and Hyaluronic Acid in Treating Knee Osteoarthritis Pain: An Updated Systematic Review and Meta-Analysis of Randomized Controlled Trials
Source: Arch Rehabil Res Clin Transl. 2026 Jan 28;8(2):100573. doi: 10.1016/j.arrct.2025.100573 (PMC13282763; doi:10.1016/j.arrct.2025.100573)
Supplement: Supplementary file 1 [file mmc1.docx]

**Supplementary Appendix 1. Search Strategy**

A comprehensive literature search was conducted in the following electronic databases: PubMed, EMBASE, Web of Science, and Scopus

#1 knee osteoarthritis[MeSH Terms] OR knee osteoarthritis[Title/Abstract] OR knee OA[Title/Abstract] OR gonarthrosis[Title/Abstract]

#2 intra-articular[Title/Abstract] OR intraarticular[Title/Abstract]

#3 platelet-rich plasma[MeSH Terms] OR platelet-rich plasma[Title/Abstract] OR PRP[Title/Abstract] OR leukocyte-rich platelet-rich plasma[Title/Abstract] OR LR-PRP[Title/Abstract]

#4 hyaluronic acid[MeSH Terms] OR hyaluronic acid[Title/Abstract] OR HA[Title/Abstract] OR viscosupplementation[Title/Abstract]

#5 randomized controlled trial[Publication Type] OR randomized[Title/Abstract] OR randomised[Title/Abstract] OR randomly[Title/Abstract] OR trial[Title/Abstract]

#6 #1 AND #2 AND (#3 OR #4) AND #5
